# Sa12b-Modified Functional Self-Assembling Peptide Hydrogel Enhances the Biological Activity of Nucleus Pulposus Mesenchymal Stem Cells by Inhibiting Acid-Sensing Ion Channels

**DOI:** 10.3389/fcell.2022.822501

**Published:** 2022-02-16

**Authors:** Letian Han, Ziyu Wang, Haoyu Chen, Jie Li, Shengquan Zhang, Sumei Zhang, Shanzhong Shao, Yinshun Zhang, Cailiang Shen, Hui Tao

**Affiliations:** ^1^ Department of Orthopedics, The First Affiliated Hospital of Anhui Medical University, Hefei, China; ^2^ Department of Spine Surgery, The First Affiliated Hospital of Anhui Medical University, Hefei, China; ^3^ Department of Clinical Laboratory, The First Affiliated Hospital of Anhui Medical University, Hefei, China; ^4^ Department of Biochemistry and Molecular Biology, School of Basic Medical Sciences, Anhui Medical University, Hefei, China

**Keywords:** intervertebral disc degeneration, acidic environment, self-assembled peptides, Sa12b, human nucleus pulposus mesenchymal stem cells, tissue engineering

## Abstract

Various hydrogels have been studied for nucleus pulposus regeneration. However, they failed to overcome the changes in the acidic environment during intervertebral disc degeneration. Therefore, a new functionalized peptide RAD/SA1 was designed by conjugating Sa12b, an inhibitor of acid-sensing ion channels, onto the C-terminus of RADA16-I. Then, the material characteristics and biocompatibility of RAD/SA1, and the bioactivities and mechanisms of degenerated human nucleus pulposus mesenchymal stem cells (hNPMSCs) were evaluated. Atomic force microscopy (AFM) and scanning electron microscopy (SEM) confirmed that RAD/SA1 self-assembling into three-dimensional (3D) nanofiber hydrogel scaffolds under acidic conditions. Analysis of the hNPMSCs cultured in the 3D scaffolds revealed that both RADA16-I and RAD/SA1 exhibited reliable attachment and extremely low cytotoxicity, which were verified by SEM and cytotoxicity assays, respectively. The results also showed that RAD/SA1 increased the proliferation of hNPMSCs compared to that in culture plates and pure RADA16-I. Quantitative reverse transcription polymerase chain reaction, enzyme-linked immunosorbent assay, and western blotting demonstrated that the expression of collagen I was downregulated, while collagen II, aggrecan, and SOX-9 were upregulated. Furthermore, Ca^2+^ concentration measurement and western blotting showed that RAD/SA1 inhibited the expression of *p*-ERK through Ca^2+^-dependent *p*-ERK signaling pathways. Therefore, the functional self-assembling peptide nanofiber hydrogel designed with the short motif of Sa12b could be used as an excellent scaffold for nucleus pulposus tissue engineering. Moreover, RAD/SA1 exhibits great potential applications in the regeneration of mildly degenerated nucleus pulposus.

## Introduction

Low back pain (LBP) is a common health problem worldwide, with a lifetime incidence rate of approximately 80% (Jensen et al., 2017). It is believed that the main cause of chronic back pain and degenerative lumbar spine disease is lumbar disc degeneration, that is, the progressive structural destruction of the intervertebral disc (IVD) (Adams and Roughley, 2006; Bailey et al., 2020). The IVD is composed of the following three parts: central gelatinous nucleus pulposus (NP), outer multilayered annulus fibrosus (AF), and cartilage endplate (Lund and Oxland, 2011). NP can provide a suitable extracellular environment for the growth and secretion of NP cells; therefore, it is one of the most critical parts of the IVD (Richardson et al., 2008). Loss of cell viability, cell proliferation, and extracellular matrix (ECM) biosynthesis is observed in the local microenvironment of IVD (Le Maitre et al., 2007; Wang et al., 2016). At present, the gold standard for intervertebral disc disease (IVDD) treatment is spinal fusion, which focuses on alleviating painful symptoms and removing the degenerated disk. However, IVDD treatment cannot reverse the progression of degeneration or restore the normal mechanical function of the IVD (Lin et al., 2016; Bian et al., 2017). Therefore, in addition to reducing the symptoms, restoring IVD function should be the focus of IVDD treatment.

Many studies have shown that reduction in the ECM components, such as collagen Ⅱ and aggrecan, secreted by nucleus pulposus cells (NPCs) play an important role in IVDD (Buckwalter, 1995; Antoniou et al., 1996; Liu et al., 2001). Several studies have focused on stimulating the regeneration of the IVD by increasing the biological activity of NPCs through the injection of growth factors (Steck et al., 2005; Shen et al., 2009; Wang et al., 2011), biological materials (Su et al., 2010; Chen et al., 2013; Mercuri et al., 2013), and cells (Meisel et al., 2006; Orozco et al., 2011; Ruan et al., 2012). Although great progress has been made on these methods, they still exhibit certain limitations when used alone. Growth factors are unable to maintain long-term biological activity because they have a short half-life. It’s insufficient biological activity of the material itself that damage to biomaterials during implantation. In addition, because of the harsh internal environment of the IVD, the survival rate of transplanted cells is very low. Therefore, a promising treatment strategy that combines the advantages of growth factors, biomaterials, and cells is required to overcome these limitations.

In recent years, tissue engineering has been successfully used in clinical orthopedics for the treatment of defects in bones (Fröhlich et al., 2010), skeletal muscles (Ricchetti et al., 2012) and tendons (Martinello et al., 2014), and damaged tissues have been replaced with biological materials and cells. Self-assembling peptide nanofiber scaffolds are promising biomaterials that have been successfully used in tissue engineering and regenerative medicine, and have become a central topic in biomaterial research (Maude et al., 2013). Scaffolds exhibit excellent biocompatibility and biological activity (Guo et al., 2007; Guo et al., 2009; Liedmann et al., 2012). RADA16-I (Ac-RADARADARADARADA16-I) is composed of a 16-residue peptide and is one of the most commonly used self-assembling nanofiber peptides (Matson and Stupp, 2012). It can produce a stable *β*-sheet structure in water and perform molecular self-assembly to form nanofibers (Zhang et al., 2005). The nanofibers then form high-order interwoven nanofiber hydrogel scaffolds under physiological conditions by alternating hydrophobic and hydrophilic amino acids (Holmes et al., 2000), resulting in the formation of ordered ECM-like hydrogels (Holmes et al., 2000; Genove et al., 2005). The cells encapsulated in self-assembling peptides can be used for three-dimensional (3D) culture precisely because of these characteristics (Zhang et al., 2005). In addition, various biologically active short peptide motifs can be easily conjugated to their C-terminus to produce growth factor analogs, which is one of the most important features of RADA16-I. These functionalized self-assembling peptide nanofiber scaffolds exhibit great potential in bone, blood vessels, and nerve regeneration (Gelain et al., 2006; Horii et al., 2007; Guo et al., 2012; Liu et al., 2013).

The IVD microenvironment is characterized by hypoxia, low pH, hypertonicity, and poor nutritional supply; therefore, with age and intervertebral disc degeneration (IDD), the internal microenvironment changes significantly (Colombier et al., 2014). A large number of studies have reported that these changes in the microenvironment have an important impact on the biological activity of cells. For example, 2% hypoxia can promote the differentiation of bone marrow-derived and adipose-derived stem cells into NPCs (Kanichai et al., 2008; Li et al., 2013). Meanwhile, low pH, hypertonicity, and low nutrition inhibit the differentiation of hNPMSCs into NPCs. However, the inhibitory effect of low pH on hNPMSCs differentiation is the most significant (Wuertz et al., 2008; Wuertz et al., 2009). RADA16-I alone lacks tissue specificity, and the biological activity of hNPMSCs is insufficient (Tao et al., 2014). Till date, there are no studies on the effect of RADA16-I on cell activity in acidic environments. It has been found that low pH inhibits the proliferation, differentiation, and ECM production of hNPMSCs through acid-sensing ion channels (ASICs) (Liu et al., 2017). Therefore, identification of a short functional polypeptide fragment of an ASIC blocker can facilitate the construction a new functional self-assembling polypeptide nanofiber scaffold material that can overcome the acidic environment in the IVD. At present, there are several blockers that can be used to regulate ASICs, such as amiloride, pctx1, apetx2, and phcrtx1; however, there are no studies on their functional short peptides. Therefore, these blockers cannot be used to construct functional self-assembling polypeptide nanofiber scaffolds.

A recent study found that Sa12b (EDVDHVFLRF), an FMRFa-related peptide extracted from the venom of the solitary wasp silver-spotted butterfly, can be used as a new blocker of ASICs. Sa12b strongly inhibited ASIC currents in rat dorsal root ganglion (DRG) neurons (Hernández et al., 2019). Sa12b exhibits great potential in restoring the activity of hNPMSCs in an acidic environment in IVDD (Wang et al., 2022). Therefore, in this study, we conjugated Sa12b to the C-terminus of RADA16-I to obtain RSA1. The peptide were then mixed with RADA16-I to form novel functionalized self-assembling peptides RAD/SA1. Finally, we evaluated the biocompatibilities and biological activity of these functionalized self-assembling peptides (RAD/SA1, RADA16-I, and RSA1) with hNPMSCs in acidic environment *in vitro*, and the mechanism was further evaluated.

## Materials and Methods

### Preparation of Self-Assembling Peptide Solutions

The RADA16-I (Ac-RADARADARADARADA16-I) and RSA1 (AC-(RADA)4-GG-EDVDHVFLRF-CONH_2_) peptides were synthesized by Sangon Biotech (purity >90%, Shanghai, China). These peptide powders were dissolved in MilliQ water at a final concentration of 1% (10 mg/ml), sonicated for 30 min, and filtered using a syringe-driven filter unit (0.22 mm HT Tuffrun membrane; Millipore). Then, the RSA1 solutions were mixed with 1% RADA16-I solution at a volume ratio of 1:1 to obtain 1% functionalized RAD/SA1 peptide mixtures.

### Scanning Electron Microscopy Assessment

All the self-assembling peptide hydrogel scaffolds and cell/scaffolds were cultured for 3 days. The samples were then fixed, dehydrated, and coated with platinum and the microstructures and cellular attachments of these scaffolds were observed using SEM. The images were captured using a GeminiSEM 300 at ×400–×20,000 magnification and 3 kv voltage.

### Atomic Force Microscopy Assessment

All peptide solutions were diluted to a working concentration of 0.01% (w/v), and 5 ml of each diluted sample was dropped onto a freshly cleaved mica surface for 25–30 s and rinsed twice gently with 100 ml distilled water. The samples were then air-dried and incubated at room temperature for 3–4 h. Images of the microstructures of the samples were acquired using AFM (Dimension Icon, United States). The scanning area was 1 × 1 μm, and the scan frequency was 1.00 Hz.

### Rheological Analysis of the Self-Assembling Peptides

For rheological analysis, 100 μL of 1% (w/v) peptide solution (RAD/SA1) was added to the parallel sample plate of the rheometer (DHR-2, United States), and allowed to stand for 30 min. The clamp spacing was set to 300 μm, and the temperature was set to 37°C. The designer’s storage (elastic) modulus (G′) and loss (viscous) modulus (G″) of the self-assembling peptides were performed within a frequency sweep range of 25–100 rad/s. The frequency sweep was performed at 37°C under a constant shear stress of 1 Pa.

### Circular Dichroism Assessment

We transferred the 25-μM peptide diluent into a quartz sample tank, and selected a path length of 0.5 cm, a wavelength range of 190–260 nm, a step length of 1 nm, and a bandwidth of 3 nm. A circular dichroic spectrum analyzer (Jasco-810-CD, Japan) was used to measure the spectra. The sample was measured thrice, the average of the three data points was determined, and the spectrum of distilled water was subtracted to obtain the final circular dichroism spectrum.

### Isolation and Culture of hNPMSCs

Table 1 provides detailed information about the age, sex, and disease status of the subjects. The study was conducted according to the criteria set by the Declaration of Helsinki. All procedures were approved by the local ethics committees of our institution and were performed with the informed consent of the patients. After washing the sample twice with phosphate-buffered saline (PBS; Gibco), the annulus fibrosus and cartilaginous endplates were carefully removed and the hNPMSCs were isolated as previously described. Briefly, the nucleus pulposus (NP) tissues were cut into approximately 1 mm^3^ pieces, digested with 0.025% collagenase II (Sigma) in a serum-free medium, incubated at 37°C with 5% CO_2_ overnight, and centrifuged at 400 × *g* for 6 min. The supernatant was removed, and the cells were resuspended and cultured in a 75-cm^2^ flask containing Dulbecco’s modified Eagle’s medium (DMEM, Gibco) with 10% fetal bovine serum (FBS; Gibco), 100 U/mL penicillin, and 100 mg/ml streptomycin. The culture medium was changed every 2–3 days.

**TABLE 1:** Sample Information.

Case NO	Age(years)	Gender	Pfirrmann grading	Segments
Case 1	23	female	IV	L5-S1
Case 2	26	male	V	L5-S1
Case 3	23	male	V	L4-L5
Case 4	26	female	IV	L4-L5

### Microscopic Observation of Cell Morphology

The culture medium was changed every three days using DMEM. Trypsin digestion and subculture was performed to observe the cell growth status, and the cell morphology was recorded daily.

### Antigen Identification

Trypsin-digested P3 generation cells were centrifuged and suspended in 100 μL PBS. Monoclonal antibodies CD34-PE, CD45-PE, HLADR-PE, CD73-PE, CD90-PE, and CD105-PE were added to each tube, and incubated for 30 min in the dark. After washing, the cells were resuspended in 0.4 ml PBS and a negative control group was maintained. Flow cytometry was used to detect the positive expression rate of cell monoclonal antibodies.

### Three-Line Differentiation Test

1) Osteogenic differentiation: P3 generation cells were seeded in a six-well plate and cultured until the cell fusion rate reached approximately 70%. The media was then replaced with osteogenic differentiation medium (Cyagen, United States) to induce cell differentiation. The plate was incubated for 21 days and the medium was changed every three days. The cells were then fixed with 4% neutral formaldehyde solution (Sangon Biotech, China) for 30 min, stained with Alizarin Red (Cyagen, United States) for 5 min, and observed under a microscope to determine the presence of calcium nodules.
2) Chondrogenic differentiation: Approximately 5 × 10^5^ P3 generation cells were collected, centrifuged, resuspended in 0.5 ml premix, and centrifuged. This procedure was repeated thrice. The cells were resuspended in 0.5 ml adult bone marrow mesenchymal stem cell chondrogenic differentiation medium (Cyagen, United States) and centrifuged at 150 × *g* for 5 min. After observing cell sedimentation, the cells were placed in a 37°C incubator containing 5% CO_2_. After 24 h, the bottom of the centrifuge tube was flicked to suspend the gathered pellets in the culture medium, and the medium was changed using 0.5 ml of complete chondrogenic differentiation medium every 2 days. After 28 days of continuous induction, the cells were stained with Alcian Blue (Cyagen, United States).
3) Adipogenic differentiation: The P3 generation cells were collected, seeded in a six-well plate, and cultured until the cell fusion rate reached 100%. The media was then switched to adipogenic differentiation medium A (Cyagen, United States), and after three days of induction, the media was switched to adipogenic differentiation medium B (Cyagen, United States). After 24 h, solution B was aspirated and the solution A was added for induction. This process was performed 3–5 times, and the culture was maintained with solution B for 4–7 days, followed by staining with Oil Red O (Cyagen, United States).

### Preparation of Media With Different pH Values

An appropriate amount of sterilized HCL (1 mol/L) and NaOH (1 mol/L) were added to the culture medium, and the pH value was monitored using a pH microelectrode (Ramagnetic phs-25, China) to prepare culture media with different pH values. After the desired pH value (6.8 or 6.2) was obtained, the medium was incubated at 37°C with 5% CO_2_ for 3 days to establish a pH balance (depending on CO_2_).

### 3D Cell Culture in Self-Assembling Peptide Scaffolds

Transwell inserts (Corning, United States) were placed in a 24-well culture plate, and 400 μL of culture medium was added to each well. hNPMSCs were suspended in 10% sucrose prior to seeding. A 20-μL aliquot of cell suspension containing 1 × 10^5^ hNPMSCs was mixed with 100 μL of the peptide solution, and the cell/peptide mixture was immediately placed in the inset. A 400-μL aliquot of culture medium was slowly added to the surface of each hydrogel piece. Gelation was allowed to proceed at 37°C for 10 min, and the medium was changed for another 30 min of incubation. The medium was changed at least twice to enhance peptide self-assembly and to equilibrate the pH. Subsequently, the medium was changed every two days.

### Cell Proliferation Assessment in an Acidic Environment

The hNPMSCs were cultured on RAD/SA1 scaffolds and medium containing Sa12b (concentration of Sa12b was 8 μg/μL at pH 6.2 and 6 μg/μL at pH 6.8) in an acidic environment, and RADA16-I was used as the control group. After 1, 3, 5, 7, and 9 days, the number of cells was evaluated using cell counting kit-8 reagents (CCK-8, Dojindo, Japan). The absorbance at 450 nm, which indirectly reflects the number of cells, was measured using a microplate reader (Elx800, Bio-Tek, United States). The experiment was performed in triplicates.

### Cell Viability Assessment

An aliquot of 1 × 10^5^ hNPMSCs was 3D-cultured in each of the self-assembling peptide hydrogel scaffolds. After one and three days, the live and dead hNPMSCs were labeled using CAM and the nucleic acid dye propidium iodide (PI, Sigma, United States), respectively. Briefly, the cells/scaffolds were incubated with 2 μmol/L CAM and 5 μmol/L PI for 30 min at room temperature in the dark, and then gently rinsed thrice using PBS. The images were collected using laser confocal microscopy, and the number of live and dead cells in five randomly selected non-overlapping areas was counted by two independent assessors. The experiment was performed in triplicates.

### Enzyme-Linked Immunosorbent Assay for ECM Secretion in an Acidic Environment

The hNPMSCs were cultured on RAD/SA1 scaffolds and medium containing Sa12b in an acidic environment, and RADA16-I was used as the control group. After culturing for 7, 14, and 28 days, the protein was extracted, and the expression levels of collagen II aggrecan, and SOX-9 in an acidic environment were detected using an ELISA kit (Colorful Gene Biological Technology Co. Ltd., China). The experiment was performed in triplicates.

### Western Blotting of ECM Secreted in the Acidic Environment

The hNPMSCs were cultured on RAD/SA1 scaffolds and medium containing Sa12b in an acidic environment, and RADA16-I was used as the control group. After culturing for 7 days and 14 days, the protein was extracted, and the expression levels of collagen II and aggrecan in an acidic environment were detected using western blotting.

### Quantitative Reverse Transcription Polymerase Chain Reaction Analysis of Gene Expression

The hNPMSCs were cultured on RAD/SA1 scaffolds and medium containing Sa12b in an acidic environment, and RADA16-I was used as the control group. After 7, 14, and 28 days, the cells were disrupted mechanically, and the total RNA was extracted using TRIzol reagent (Invitrogen, United States), according to the manufacturer’s instructions. Complementary DNA was synthesized from total RNA using a reverse transcription reagent (SYBR Premix Ex Taq, Takara, Japan). The experiment was performed in triplicates.

The expression levels of the hNPMSCs-related genes (encoding collagen I, collagen II, aggrecan and SOX-9) were analyzed using qRT-PCR, and glyceraldehyde-3-phosphate dehydrogenase (GAPDH) housekeeping gene was used as the control. The primers for all of the genes were designed using Premier 5.0, and are listed in Table 2. A SYBR Premix Ex Taq PCR kit (Takara, Japan) and Mini OpticonTM Detector System (Bio-Med, United States) were used for qRT-PCR analysis. After an initial denaturation at 95°C for 20 s, 40 cycles of PCR amplification were performed at 95°C for 5 s and 60°C for 20 s. Then, a cycle threshold (Ct) value was obtained for each sample, and an average value of the experiment performed in triplicates was calculated. The 2^−ΔΔCt^ values were used to evaluate the relative expression levels of these genes. All data were acquired in three independent experiments, in which each sample was evaluated in triplicates.

**TABLE 2:** Primers used in qRT-PCR.

Genes	Sense primer	Antisense primer
GAPDH	GAAGGTCGGAGTCAACGG	GGA​AGA​TGG​TGA​TGG​GAT​T
Aggrecan	ACG​GCT​TCT​GGA​GAC​AGG​ACT​G	CTG​GGA​TGC​TGG​TGC​TGA​TGA​C
SOX-9	AGG​AGA​GCG​AGG​AGG​ACA​AGT​TC	TGT​TCT​TGC​TGG​AGC​CGT​TGA​C
Collagen II	GGA​GCA​GCA​AGA​GCA​AGG​AGA​AG	TCA​TCT​GGA​CGT​TGG​CAG​TGT​TG
Collagen I	CCT​GGA​AAG​AAT​GGA​GAT​GAT​G	ATC​CAA​ACC​ACT​GAA​ACC​TCT​G

### Evaluation of p-ERK Expression in Cells in an Acidic Environment

The hNPMSCs were cultured on RAD/SA1 scaffolds and medium containing Sa12b in an acidic environment, and RADA16-I was used as the control group. The protein was extracted, and the expression levels of *p*-ERK in an acidic environment were detected using western blotting.

### Evaluation of the Amount of Ca^2+^ in Cells in an Acidic Environment

The hNPMSCs were cultured on RAD/SA1 scaffolds and medium containing Sa12b in an acidic environment, and RADA16-I was used as the control group. The Ca^2+^ kit (Dojindo Laboratories, Japan) was used to stain each group of cells, and a confocal microscope was used to detect the amount of Ca^2+^ in the cells.

### Statistical Analysis

All data were statistically analyzed using SPSS 23.0 and are presented as the mean values ±standard deviation. An analysis of variance (ANOVA) of factorial design was performed to analyze the main effect and the interaction between the groups and the time periods. A one-way ANOVA was performed for multiple-group comparisons, and the Student−Newman−Keuls test (homogeneity of variance) or Tamhane’s test (heterogeneity of variance) was performed to compare any two groups. *p* < 0.05 was considered a significant difference.

## Results

### Formation of Hydrogel-Like Materials by New Self-assembling Peptides and Survival of hNPMSCs.

**FIGURE 1:**
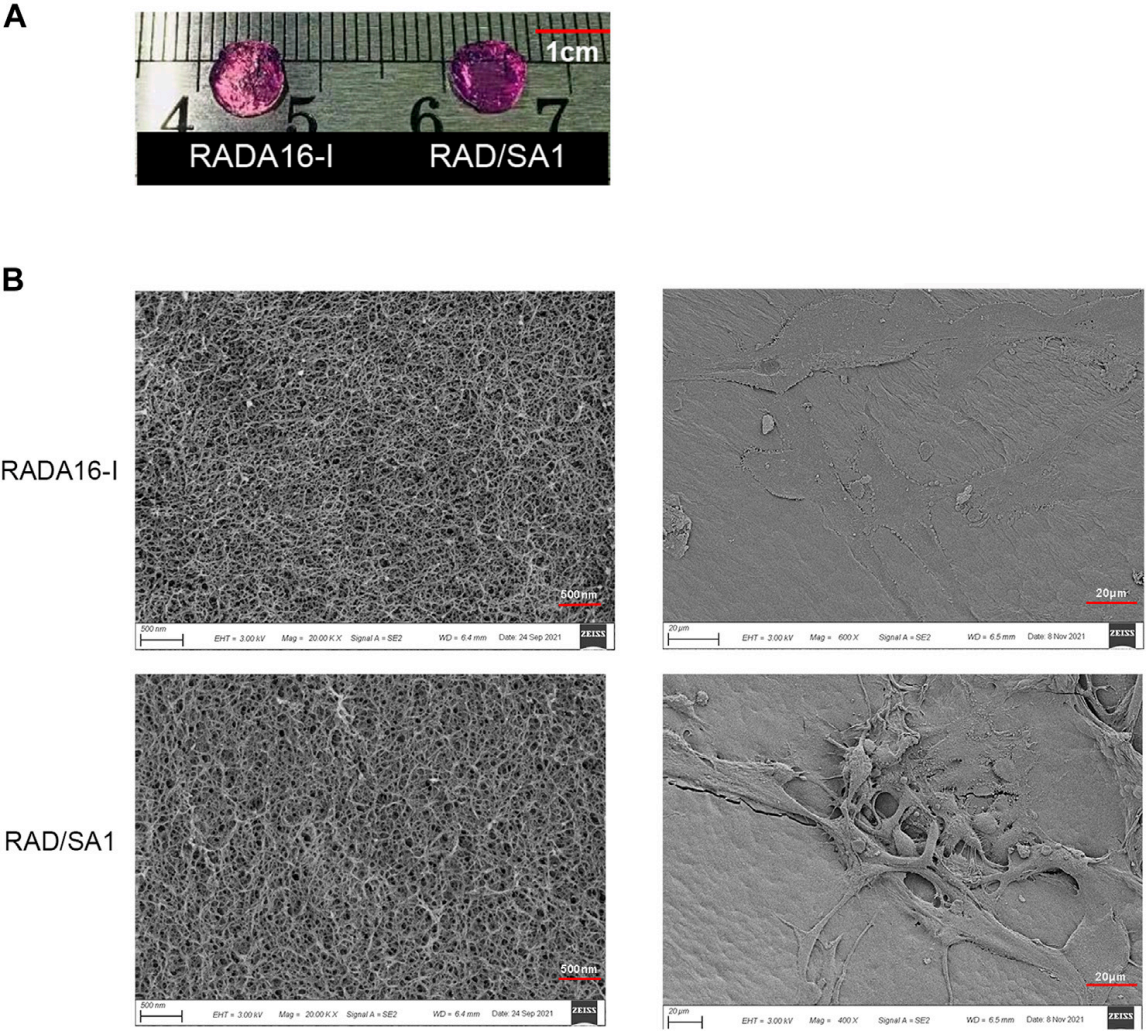
**(A)** Both RADA16-I and RAD/SA1 formed a transparent hydrogel-like material at 37°C *in vitro*. **(B)** The SEM images showed that all of the designer self-assembling peptides had formed nanofibers that had interwoven to form porous structures and that the hNPMSCs had attached to the nanofibers by extending many pseudopodia.

Sa12b was conjugated to the C-terminus of RADA16-I using solid-phase synthesis to construct a functionalized self-assembling polypeptide, RSA1. Both RADA16-I and RAD/SA1 formed a transparent hydrogel-like material at 37°C *in vitro* (Figure 1A) with no significant differences in appearance; conversely, RSA1 failed to form a hydrogel-like material.

The self-assembling polypeptide RADA16-I and functionalized self-assembling polypeptide RAD/SA1 were prepared into hydrogel scaffold materials *in vitro*, and their ultrastructure was observed using SEM. The results showed that all peptides could form a large number of nanofibers *in vitro* and interweave into a staggered and ordered pore-like structure scaffold material (Figure 1B). The nanofiber diameter was approximately 10–40 nm, the pore diameter was approximately 5–200 nm, and its water content exceeded 99%. The ultrastructure of the ECM of natural intervertebral discs has similar features. In the new hydrogel scaffold material, a large number of hNPMSCs were tightly adhered to the nanofibers of the scaffold material by extending several pseudopodia-like structures, and their shape presented a 3D structure.

**FIGURE 2:**
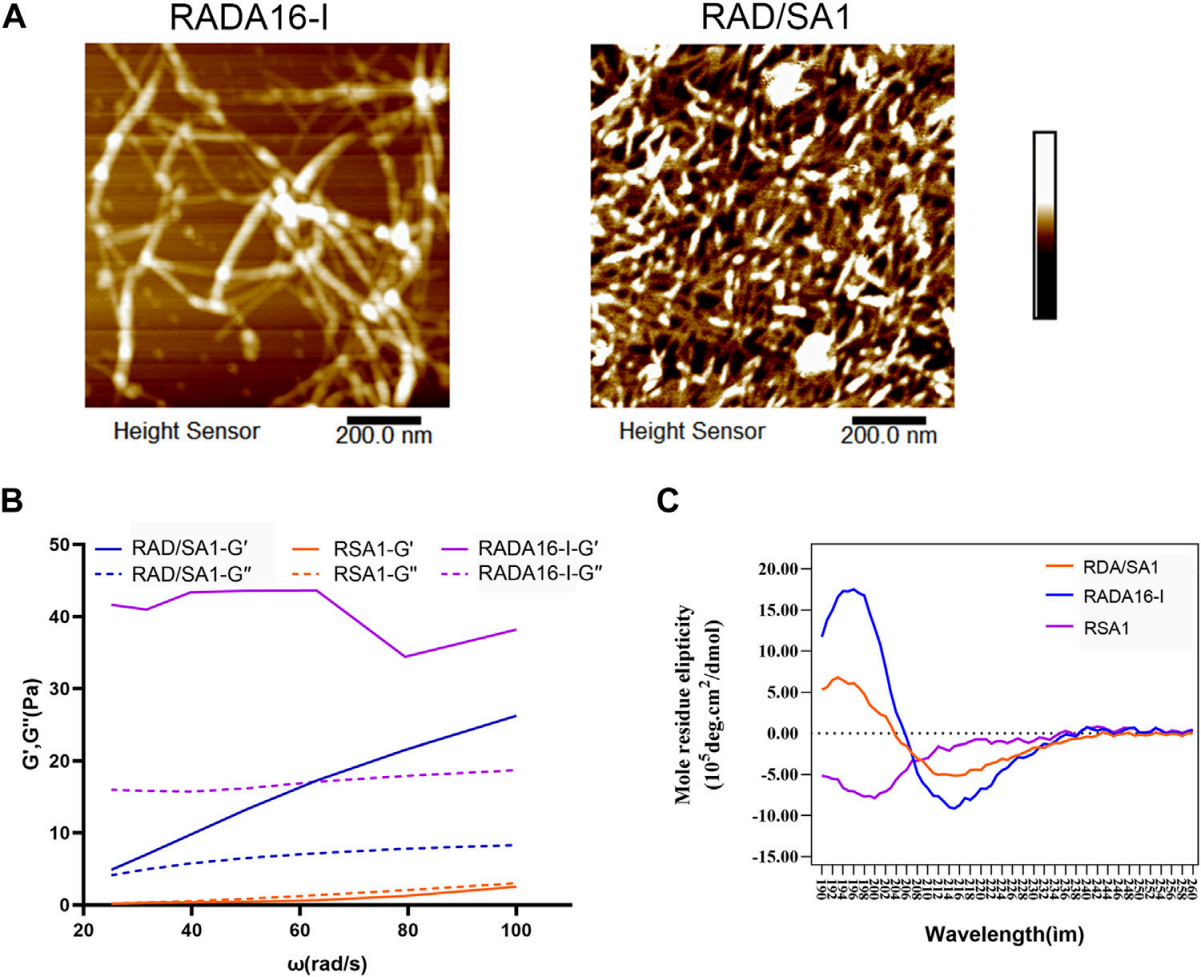
**(A)** The AFM results showed that nanofiber formation occurred in of all of the solutions of the self-assembling peptides. **(B)** Results of the oscillation frequency sweep data on 1% (w/v) of the designer self-assembling peptides with DMEM at 37°C. A typical gel-like behavior was evident as the storage modulus values of the RAD16-I and RAD/SA1 were higher than the loss modulus values in the range of the shear rate frequencies. **(C)** The functionalized self-assembling polypeptide mixed with RADA16-I in equal proportions resulted in a typical *β*-sheet structure.

As shown in Figure 2A, AMF revealed that RADA16-I and functionalized self-assembling peptides formed several nanofibers, ranging in length from a few hundred nanometers to micrometers. However, the diameter of the nanofibers assembling from a mixture of functionalized peptides was larger than that of pure RADA16-I. RSA1 failed to form nanofibers. The diameters of nanofibers in RADA16-I and RAD/SA1 solutions are approximately 10–40 nm.

The frequency sweep results obtained at 37°C showed that the viscoelasticity of this hydrogel was similar (including storage modulus and loss modulus values), and both G′ and G″ were independent of the shear rate frequency (Figure 2B). The typical gel-like behavior was observed and in the shear rate frequency range, the storage modulus (G′, 100 Pa) of this new type of hydrogel was greater than that of the loss modulus (G″, 100 Pa).

The results of circular dichroism spectroscopy (Figure 2C) showed that the self-assembling peptide RADA16-I formed a negative peak at 216 nm and a positive peak at 196 nm, indicating a typical *β*-sheet structure. Conjugation of the short functional fragment of Sa12b to the C-terminus of RADA16-I did not result in peak formation in the circular dichroic spectrum of the functionalized self-assembling polypeptide RSA1, indicating a free bond structure. The functionalized self-assembling polypeptide mixed with RADA16-I in equal proportions resulted in a typical *β*-sheet structure; however, the absolute peak value of RAD/SA1 was smaller than that of RADA16-I.

### Identification of hNPMSCs

**FIGURE 3:**
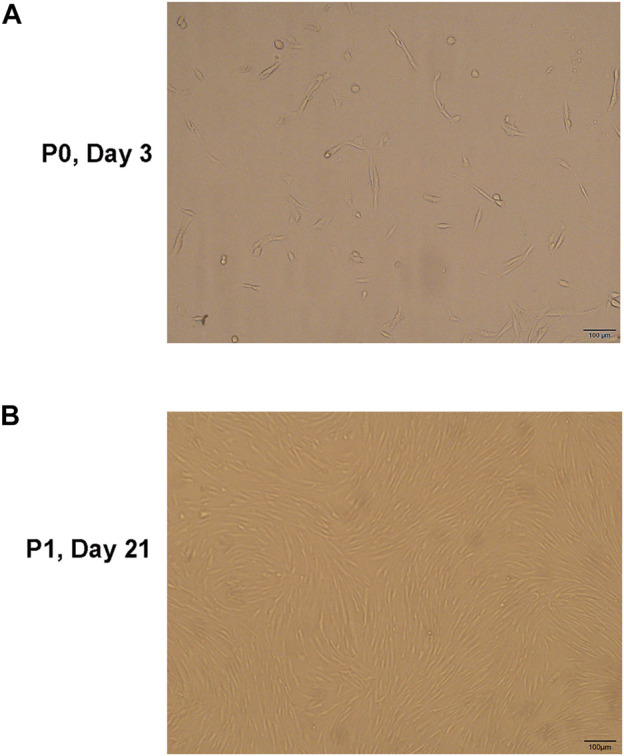
**(A)** The hNPMSCs exhibited a short, rod-like shape in primary culture. **(B)** The hNPMSCs showed uniform spindle filament spiral growth after subculture.

After the primary cells were cultured for 3–4 days, they adhered to the flask and appeared spindle- and vortex-shaped under the microscope. After approximately 20 days of culture, the cells fused, and fusion increased rapidly after passage. The cell morphology of P2 and P3 generations remained uniform and grew into spindle- and vortex-shaped cells (Figure 3).

**FIGURE 4:**
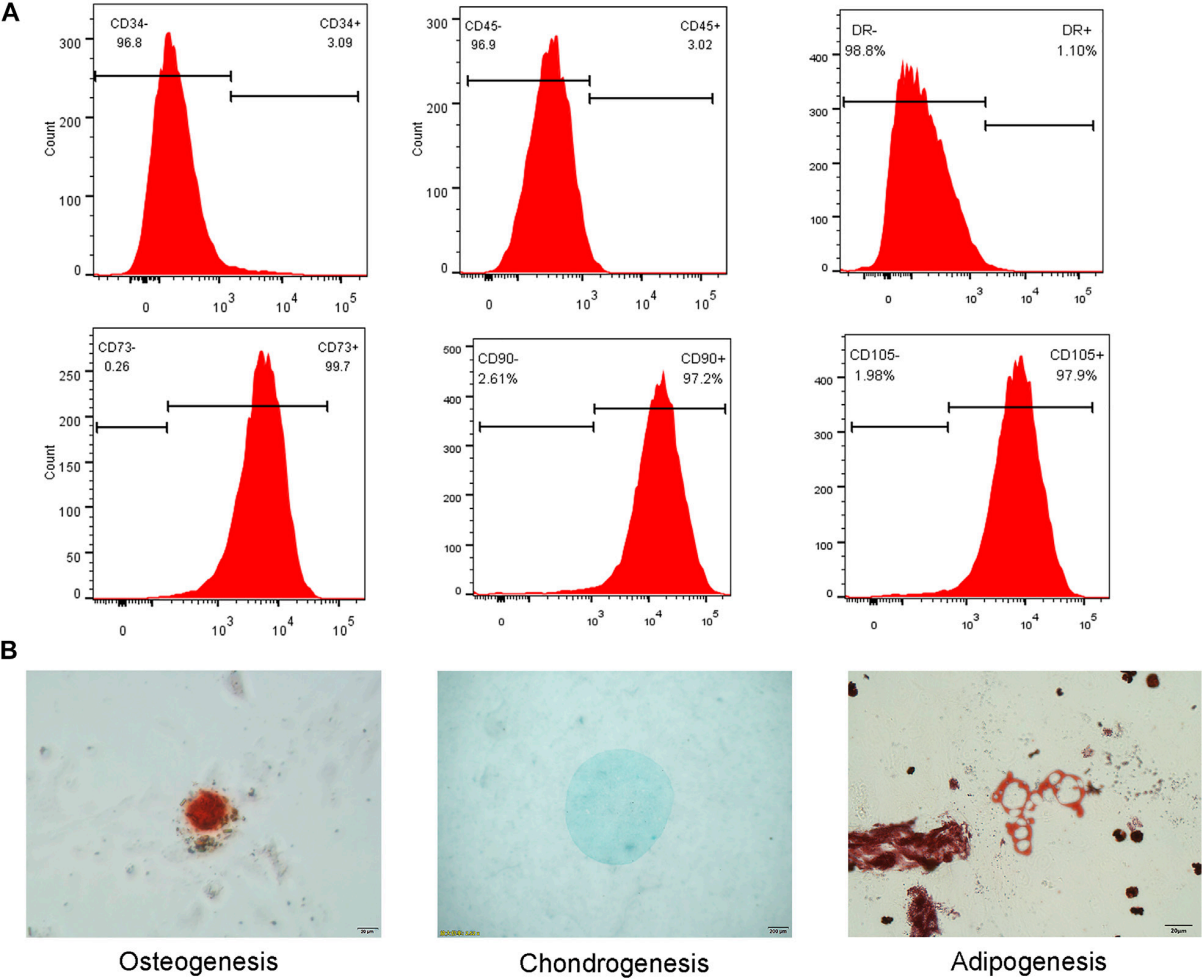
**(A)** Flow cytometry analysis showed low expression of CD34, CD45 and HLA-DR, and high expression of CD73, CD90 and CD105. **(B)** Three lineage differentiation of hNPMSCs. Alizarin red staining of calcium nodules indicated osteogenic differentiation. Alcian blue staining of cartilage indicated chondrogenic differentiation. Oil red O staining of intracellular lipid vacuoles indicated adipogenic differentiation.

Flow cytometry analysis revealed low expression of CD34 (3.09%), CD45 (3.02%) and HLA-DR (1.10%), and high expression of CD73 (99.7%), CD90 (97.2%) and CD105(97.9%) in the extracted cells (Figure 4A).

Osteogenic differentiation and culture of P3 generation cells resulted in the formation of punctate opaque calcium nodules on the cell surface, which increased with culture time. After 21 days of induction, red calcium nodules were observed upon Alizarin red staining (Figure 4B). Chondrogenic differentiation of P3 cells was observed, and the microspheres exhibited a milky white mass after approximately one week of culture. After induction for 28 days, the white masses were fixed with 4% paraformaldehyde and embedded in paraffin. Alcian Blue staining revealed an evenly blue-stained ECM (Figure 4B). Adipogenic differentiation of P3 generation cells resulted in small lipid droplets that appeared in the cytoplasm. Oil Red O staining revealed that the lipid droplets became large and round after 28 days (Figure 4B).

### Biological Activity of hNPMSCs in an Acidic Environment

**FIGURE 5:**
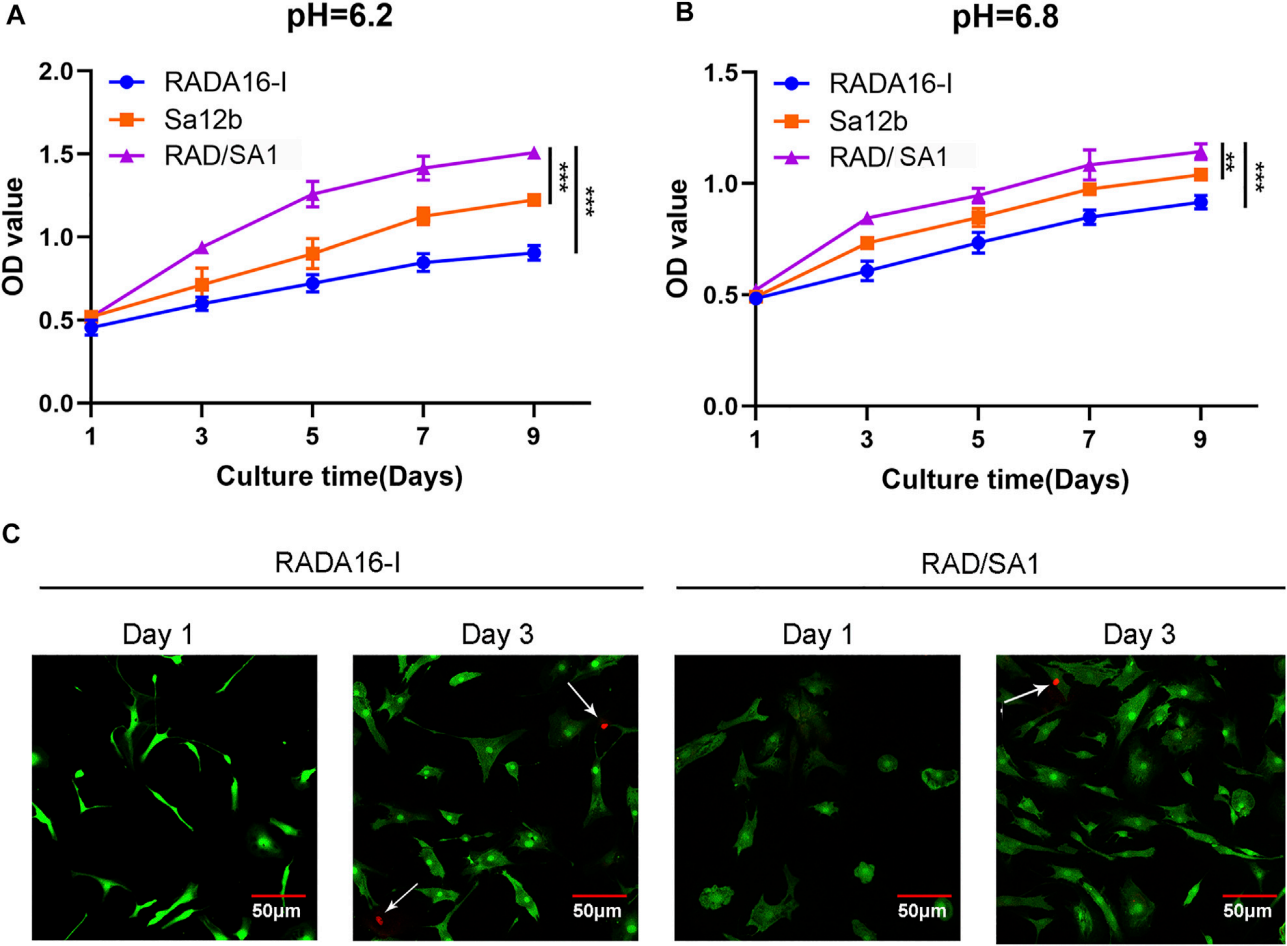
CCK-8-based quantification analysis demonstrated that the amount of hNPMSCs 3D cultured in RAD/SA1 were significantly higher than those cultured in Sa12b and RADA16-I for 1 day, 3, 5, 7 and 9 days, respectively. When the two groups compared, ** indicates *p* < 0.01 and *** indicates *p* < 0.001. **(A)** hNPMSCs were cultured at pH 6.2. **(B)** hNPMSCs were cultured at pH 6.8. **(C)** Live and dead cell viability assay for hNPMSCs that were 3D cultured in RADA16-I and RAD/SA1 for 1 and 3 days, respectively. The green fluorescent cells are the live cells labeled with CAM, and the red fluorescent cells indicated by white arrows are dead cells labeled with PI.

The hNPMSCs were incorporated into the functionalized self-assembling polypeptide RAD/SA1 hydrogel and cultured with Sa12b for 1, 3, 5, 7, and 9 days. The CCK-8 experiment was performed using RADA16-I as a control. Assessment of cell proliferation ability under the two pH (*pH* = 6.2 and 6.8) environments revealed that the cell proliferation rate in the functionalized self-assembling polypeptide hydrogel scaffold material RAD/SA1 was significantly higher than that in the pure self-assembling polypeptide RADA16-I and Sa12b group(*p* < 0.01) (Figures 5A,B). The number of degenerated hNPMSCs gradually increased with an increase in culture time, and the interaction effect between grouping and culture time was statistically significant (*p* < 0.01).

The hNPMSCs were incorporated on the surface of the self-assembling polypeptide RADA16-I and functionalized self-assembling polypeptide RAD/SA1 hydrogel, and cultured for one and three days. Cytotoxicity testing of the scaffold material revealed that a small number of PI red-stained dead cells were visible inside the two groups of polypeptide hydrogel scaffold materials, and there was no significant difference between the groups (Figure 5C). The number of dead cells did not increase significantly with an increase in culture time. These results indicated that the functionalized self-assembling polypeptide nanofiber scaffold material did not increase the cytotoxicity of the self-assembling polypeptide hydrogel.

**FIGURE 6:**
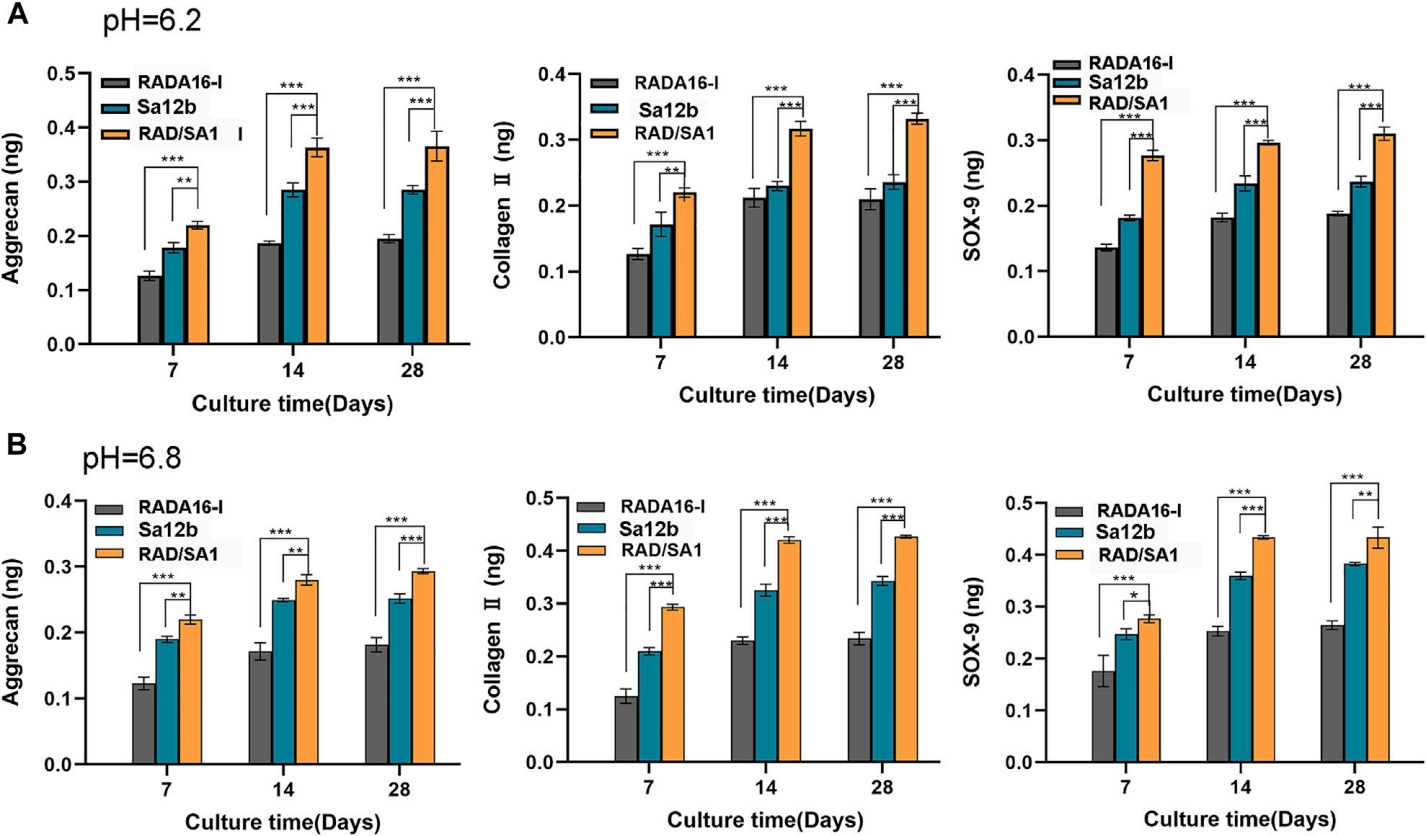
ELISA revealed that the amounts of collagen II, aggrecan and SOX-9 secreted by hNPMSCs were significantly different among the three groups (RAD/SA1, Sa12b and RADA16-I) after 7, 14 and 28 days, respectively. **(A)** hNPMSCs were cultured at pH 6.2. **(B)** hNPMSCs were cultured at pH 6.8. When the two groups compared, ** indicates *p* < 0.01, and *** indicates *p* < 0.001.

The hNPMSCs were incorporated in the functionalized self-assembling polypeptide RAD/SA1 hydrogel and 3D-cultured with Sa12b for 7, 14, and 28 days. RADA16-I was used as the control. ELISA revealed that the amounts of collagen II, aggrecan, and SOX-9 secreted by degenerated hNPMSCs were significantly different at pH 6.2 and pH 6.8 (*p* < 0.05) (Figure 6). The amounts of collagen II, aggrecan, and SOX-9 secreted by degenerated hNPMSCs at different culture time points also showed significant differences (*p* < 0.05). Moreover, the interaction effect between grouping and culture time was statistically significant for the expression of collagen II, aggrecan, and SOX-9 (*p* < 0.05). When compared with the pure self-assembling polypeptide RADA16-I hydrogel scaffold material, the RAD/SA1 hydrogel scaffold material effectively secreted degenerative hNPMSCs collagen II, aggrecan, and SOX-9 (*p* < 0.05). Furthermore, the functionalized self-assembling polypeptide hydrogel scaffold materials did not promote the secretion of collagen II, aggrecan, or SOX-9 with an increase in culture time. This change occurred 14 days prior to culture and was maintained for at least 28 days hNPMSCs were incorporated in the RAD/SA1 hydrogel and 3D-cultured with Sa12b for 7 and 14 days. RADA16-I was used as the control.

**FIGURE 7:**
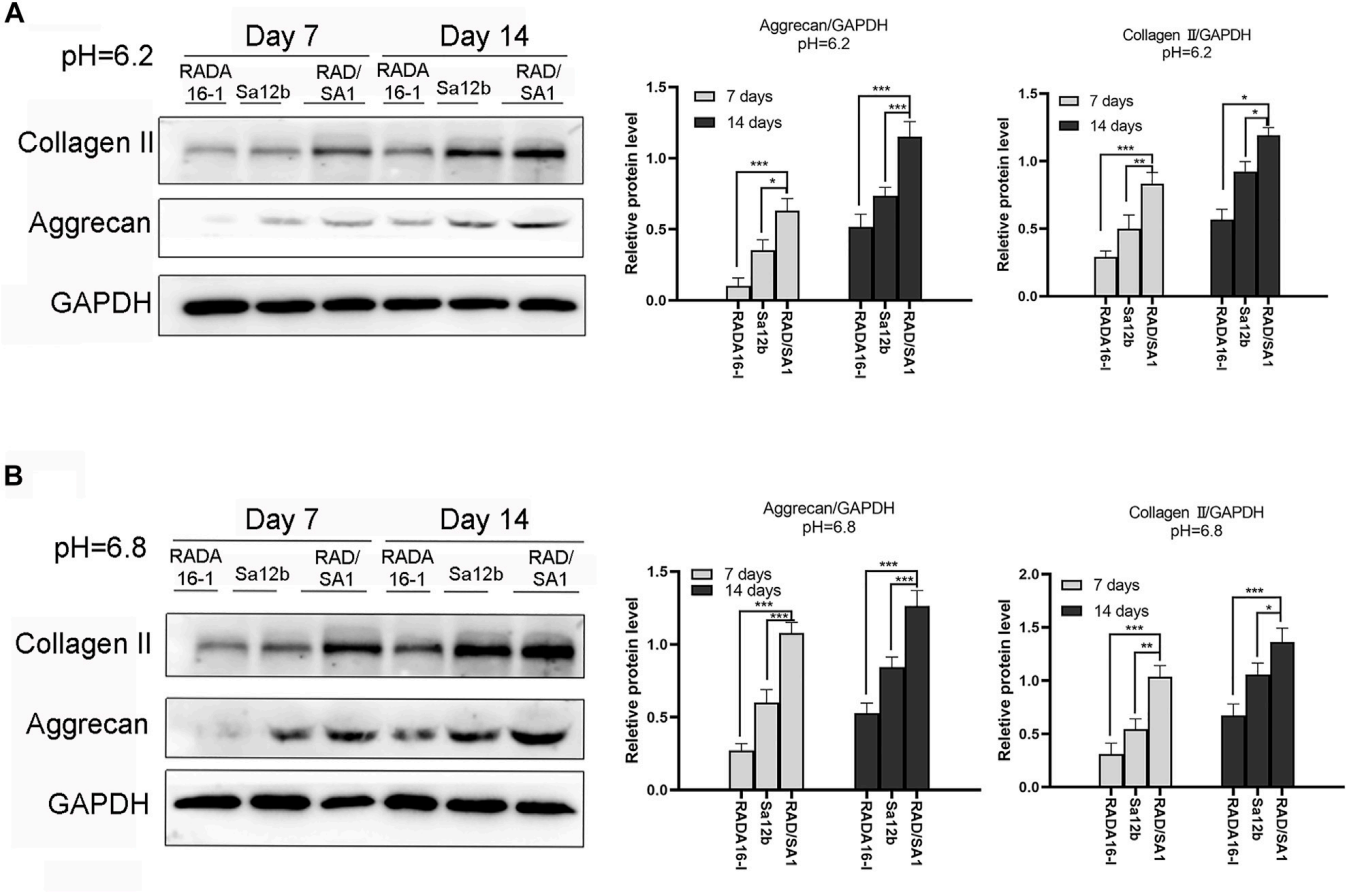
Western blotting revealed that the amounts of collagen II and aggrecan secreted by hNPMSCs were significantly different among the three groups (RAD/SA1, Sa12b and RADA16-I) after 7 and 14 days, respectively. **(A)** hNPMSCs were cultured at pH 6.2. **(B)** hNPMSCs were cultured at pH 6.8. When the two groups compared, * indicates *p* < 0.05, ** indicates *p* < 0.01, and *** indicates *p* < 0.001.

Western blotting was used to analyze the ability of hNPMSCs to secrete collagen II and aggrecan (Figure 7). The results indicated that the amount of collagen II and aggrecan secreted by hNPMSCs in the two scaffold materials were different at pH 6.2 and pH 6.8. When compared with the pure self-assembling polypeptide RADA16-I hydrogel scaffold material and pure Sa12b, the functional self-assembling polypeptide RAD/SA1 hydrogel scaffold material effectively promoted the secretion of collagen II and aggrecan by hNPMSCs, which increased with culture time (7 and 14 days).

**FIGURE 8:**
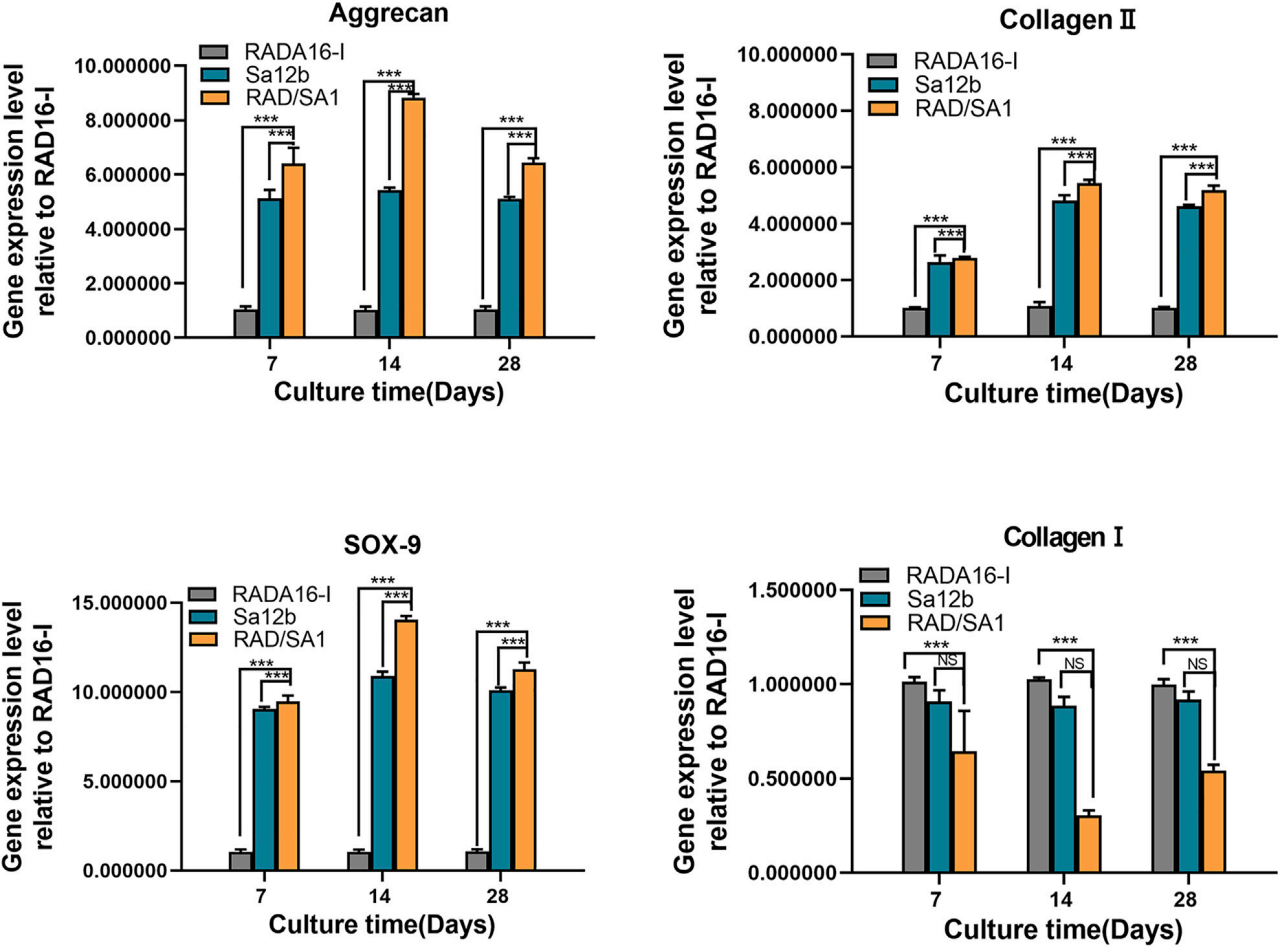
QRT-PCR analysis revealed that the mRNA expression of collagen I, collagen II, aggrecan, and SOX-9 secreted by hNPMSCs were significantly different among the three groups (*pH* = 6.2) after 7, 14 and 28 days, respectively. When the two groups compared, * indicates *p* < 0.05, ** indicates *p* < 0.01, and *** indicates *p* < 0.001.

**FIGURE 9:**
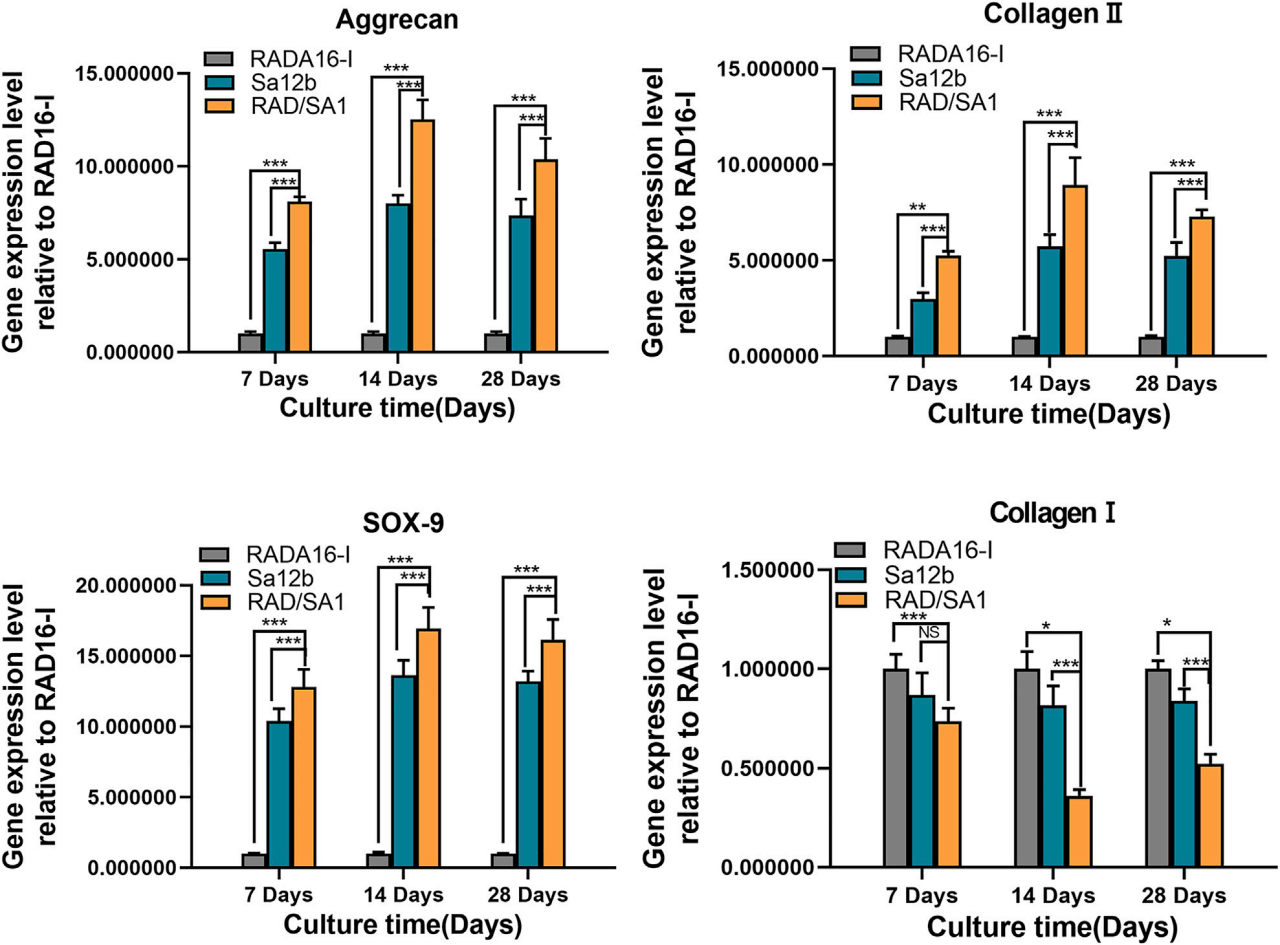
QRT-PCR analysis revealed that the mRNA expression of collagen I, collagen II, aggrecan, and SOX-9 secreted by hNPMSCs were significantly different among the three groups (*pH* = 6.8) after 7, 14 and 28 days, respectively.When the two groups compared, * indicates *p* < 0.05, ** indicates *p* < 0.01, and *** indicates *p* < 0.001.

Furthermore, qRT-PCR analysis revealed that the mRNA expression of collagen I, collagen II, aggrecan, and SOX-9 secreted by hNPMSCs was significantly different among the three groups (Figures 8, 9). When compared with the pure self-assembling polypeptide RADA16-I and pure Sa12b, the functional self-assembling polypeptide hydrogel scaffold material RAD/SA1 significantly increased the expression of collagen II, aggrecan, and SOX-9, and significantly reduced the expression of collagen I (*p* < 0.05).

### Apoptosis of hNPMSCs in an Acidic Environment Through the Ca^2+^/P-ERK Pathway

**FIGURE 10:**
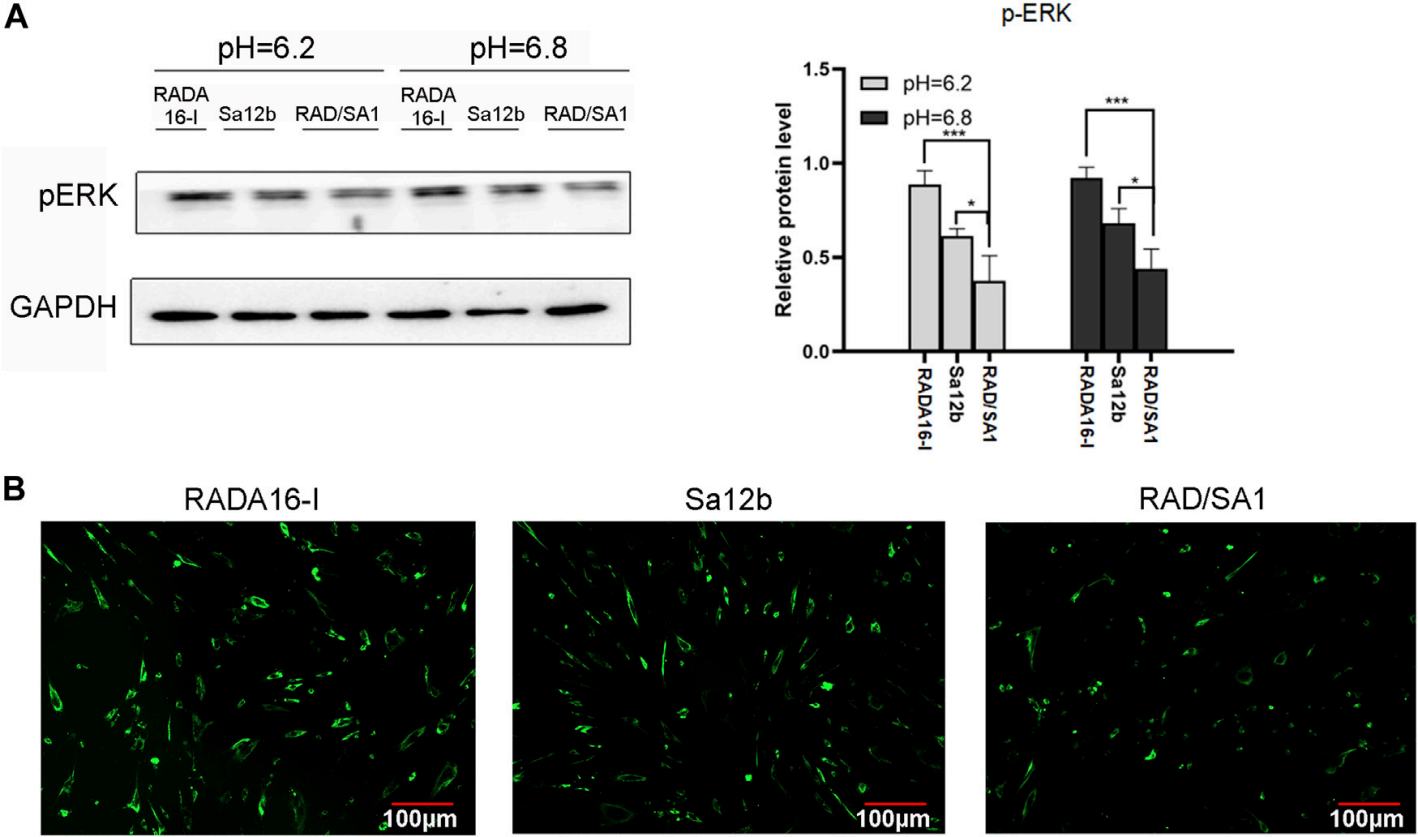
**(A)** Western blotting revealed that the protein expression of *p*-ERK receptor in RAD/SA1 was significantly lower than that in the other two groups, and the control group had the highest expression level. When the two groups compared, * indicates *p* < 0.05, ** indicates *p* < 0.01 and *** indicates *p* < 0.001. **(B)** The Ca^2+^ levels in the three groups of cells were measured using a Ca^2+^ kit. The results showed that the Ca^2+^ concentration in RADA16-I was higher than that in the other two groups, and the Ca^2+^ content in RAD/SA1 was the lowest.

P-ERK is widely regarded as an important indicator of apoptosis. To check whether *p*-ERK phosphorylation was regulated by Sa12b, we performed western blotting analysis and found that the protein expression of *p*-ERK receptor in RAD/SA1 was significantly lower than that in the other two groups, and the control group had the highest expression level (Figure 10A) (*p* **<** 0.05). Therefore, the Sa12b functional fragment reduced the level of *p*-ERK when compared with that in the control group.

hNPMSCs were incorporated in the functionalized self-assembling polypeptide RAD/SA1 hydrogel and 3D-cultured with Sa12b. RADA16-I was used as the control. The Ca^2+^ levels in the three groups of cells were measured using a Ca^2+^ kit. The results showed that the Ca^2+^ concentration in RADA16-I was higher than that in the other two groups, and the Ca^2+^ content in RAD/SA1 was the lowest (Figure 10B). Therefore, when compared with the control group, the Sa12b functional fragment reduced the amount of intracellular Ca^2+^.

## Discussion

Tissue engineering strategies are among the most promising methods for the treatment of IDD (Leung et al., 2011). The ideal biomaterial scaffold for tissue engineering must possess the following characteristics (Yang and Li, 2009; Francisco et al., 2013): 1) good biocompatibility, 2) good biological activity, 3) injectability, 4) *in situ* gelation. Previous studies have found that the self-assembling polypeptide RADA16-I has good biocompatibility and stimulates the biological activity of hNPMSC. Moreover, *in situ* gelation can be achieved using minimally invasive techniques, indicating broad clinical application. However, RADA16-I alone lacks tissue specificity, and the biological activity for NP cells is insufficient (Tao et al., 2014). Therefore, in this study, we used solid-phase synthesis to conjugate the short functional motif of Sa12b to the carbon end of RADA16-I to improve the biological activity of hNPMSCs in an acidic environment. The results indicated that functionalized self-assembling peptides could not only self-assemble into nanofiber hydrogel scaffolds but also promote the proliferation of human hNPMSCs and the biosynthesis of ECM in an acidic environment. These results indicate that the functionalized self-assembling peptide nanofiber hydrogel can be used as an ideal scaffold material for hNPMSCs regeneration in acidic environments.

Previous studies have reported that the nanofiber formation ability, self-assembly performance, and secondary structure of RADA16-I may be affected by the insertion of short functional fragments; however, this effect can be mitigated by shortening the insertion (Zhang et al., 2005; Gelain et al., 2006; Horii et al., 2007). In this study, we found that RSA1 failed to form *β*-sheets and nanofibers after inserting a short functional fragment, Sa12b, into the C-terminus of RADA16-I. The short functional fragment Sa12b can significantly affect the material properties of the self-assembling polypeptide RADA16-I. In addition, a typical *β*-sheet structure with nanofibers was formed and rapidly self-assembling into a hydrogel scaffold material *in vitro* when RSA1 was mixed with RADA16-I in an equal volume. This new hydrogel scaffold material can reduce the effect of functionalized self-assembling peptides containing short functional fragments of Sa12b on the properties of RADA16-I materials.

The NP tissue selected in this study was obtained from patients with lumbar disc herniation and belonged to the degeneration intervertebral disc (Pfirrmann grades IV-V). Isolation and *in vitro* culturing revealed adherent vortex-shaped cells, with low expression of CD34, CD45 and HLA-DR and high expression of CD73, CD90, and CD105. These cells also exhibited the ability to differentiate into osteocytes, adipocytes, and chondrocytes. Therefore, based on the standard evaluation of the International Stem Cell Therapy Association (Dominici et al., 2006), the isolated and cultured cells were hNPMSCs.

The 3D environment is very important for cell viability. In this study, we found that RAD/SA1 can form an ECM-like structure under physiological conditions. The microstructure confirms that the new peptide can form several long and high-order interwoven 3D nanofiber networks with a fiber diameter of 10–40 nm, a pore range of 5–200 nm, and an extremely high water content (>99%). These nanofiber structures were significantly smaller than high-density nanoparticles, which mimics a natural 3D microenvironment, such as an ECM. In addition, the nanofiber network was conducive to the diffusion of nutrients and excretion of metabolites. Due to the lack of nutrition in NP, this aspect is particularly important for NP engineering applications. In addition, the new peptide can spontaneously form a NP tissue-like hydrogel scaffold under physiological conditions, which indicates that the RAD/SA1 solution can be injected into NP by a minimally invasive method and can self-assemble into a nanofiber hydrogel scaffold *in vivo*.

The biocompatibility of biomaterials is another important aspect in tissue engineering. In our study, we evaluated the biocompatibility of RADA16-I-based peptide hydrogel scaffolds for NP tissue engineering. The hNPMSCs cultured in a peptide solution at 37°C successfully formed a hydrogel scaffold. We observed that high-density hNPMSCs could adhere to all the designed peptides by extending pseudopodia-like structures to the nanofibers of the hydrogel scaffold. The cell viability experiment results confirmed that the functionalized self-assembling polypeptide nanofiber scaffold material did not increase the toxicity of RADA16-1 to hNPMSCs. Therefore, this new type of hydrogel scaffold provides hNPMSCs with reliable biocompatibility.

The functional short motif Sa12b used in this study is an FMRFa-related peptide extracted from organisms. Studies have found that Sa12b has a strong inhibitory effect on ASIC currents in DRG neurons (Hernández et al., 2019). In this study, the results showed that Sa12b binds to the C-terminus of RADA16-I and provides bioactive stimulation for hNPMSCs in an acidic environment in the 3D hydrogel scaffold. When compared with the RADA16-I group, our results showed that the hydrogel scaffolds containing Sa12b functionalized peptides increased the proliferation of hNPMSCs in an acidic environment and upregulated the expression of collagen Ⅱ, SOX-9, and aggrecan. IVD is essential for maintaining its function in acidic environments, and Sa12b promotes the biological activity of cells. These findings indicate that the functional short peptide motif Sa12b derived from FMRFa-related peptides contributes to the biological activity of mesenchymal stem cells in an acidic environment *in vitro* and is essential for NP tissue regeneration.

To further evaluate the ability of RAD/SA1 to promote the biological activity of hNPMSCs, we cultured hNPMSCs in RADA16-I as a blank control group (BCG) and in a medium containing Sa12b as a positive control group (PCG). The results showed that the proliferation rate and ECM secretion level of hNPMSCs in PCG were significantly higher than those in the BCG. These results indicated that acid environment could inhibit the biological activity of hNPMSCs while Sa12b could improve the cell damage caused by the acid environment. After conjugating the functionalized peptide of Sa12b onto the C-terminus of RADA16-I, the bioactivities of the novel functionalized self-assembling peptide were remarkable advanced. The proliferation rate and ECM secretion level of hNPMSCs in RAD/SA1 were significantly higher than those both in the PCG and BCG. We speculated that the higher proliferation rate of hNPMSCs grown in the functionalized self-assembling peptide hydrogel in an acidic environment and the high secretion level of ECM were majorly due to the release of Sa12b to overcome the acidic environment and providing an easy-to-pass 3D environment by the self-assembling scaffold.

Although the biological activities of hNPMSCs in Sa12b were significantly higher than those in BCG, Sa12b was added to the culture medium every two days during the 28-day period because of its short half-life. Therefore, in order to maintain the biological activity of Sa12b in clinical applications, Sa12b must be injected multiple times into the IVD. However, it is unrealistic. On the other hand, in this study, we found that the biological activities of Sa12b can be maintained, at least for 28 days *in vitro*, by conjugating its short functional motif onto the C-terminal of RADA16-I. The reason for this might be that the chemical bonds (β-sheet) self-assembling to form a nanofiber hydrogel scaffold under physiological conditions were significantly stronger than those formed by traditional physical mixing method. Thus, the functional motifs Sa12b could be slowly released to increase the ECM secretion rate for at least 28 days *in vitro*. Moreover, the functionalized self-assembling peptide RAD/SA1 has superior clinical application potential than Sa12b.

To verify whether Sa12b inhibits the cell damage due to the acidic environment through the Ca^2+^/p-ERK pathway, we used the Ca^2+^ kit to detect the Ca^2+^ concentration in each group of cells, followed by western blotting to detect the expression of the *p*-ERK protein. We found that the intracellular Ca^2+^ concentration in the RAD/SA1 group was lower than that in the other two groups, and was the highest in the blank control group. The expression of *p*-ERK was the lowest in the RAD/SA1 group and the highest in the blank control group. These results indicate that Sa12b reduces the intracellular Ca^2+^ content as well as the expression of *p*-ERK. This is consistent with the results of other studies (Sun et al., 2018; Guo et al., 2019), Therefore, Sa12b can influence the biological activity of hNPMSCs in an acidic environment through Ca^2+^/p-ERK.

Although the functionalized self-assembling polypeptide nanofiber hydrogel scaffold material containing the short functional fragment of Sa12b exhibited good biocompatibility and biological activity for hNPMSCs in an acidic environment, the functionalized self-assembling polypeptide hydrogel scaffold materials exhibit poor biomechanical properties, which hinders potential clinical application (Reitmaier et al., 2012; Koutsopoulos and Zhang, 2013). The spine has strong axial pressure, which makes the purely functional self-assembling peptide hydrogel scaffold material unbearable. The IVD is a complex anatomical structure consisting of the central NP, peripheral fibrous annulus, and upper and lower endplates. The IVD alleviates the axial direction of the spine by changing the osmotic pressure in NP only when the peripheral fibrous annulus structure is complete (Antoniou et al., 1996; Adams and Roughley, 2006). Studies have shown that when the peripheral fibrous annulus is intact, even simple implantation of autologous bone marrow mesenchymal stem cells can repair the early and mid-stage IDD (Orozco et al., 2011). Therefore, functionalized self-assembling peptide hydrogel nanofiber scaffold materials are the same as other biological treatment methods, which are more suitable for the early and mid-stage IVDD, such as Pfirrmann grade II or III, when the structure of the annulus is still intact. However, this study has the following limitations: 1) we did not conduct *in vivo* experiments. An *in vitro* culture is different from *in vivo* conditions, and additional systemic reactions may occur under *in vivo* conditions. 2) A detailed investigation of the mechanism of action is required. Therefore, although the novel hydrogel is suitable for the treatment of clinical diseases, it is necessary to conduct further research to evaluate the effect of the material.

## Conclusion

In this study, we firstly combined the functional short peptide Sa12b onto the C-terminus of RADA16-I, and then mixed with RADA16-I to obtain a novel functionalized self-assembling peptide RAD/SA1. The results demonstrated that the novel functionalized self-assembling peptide hydrogel scaffold RAD/SA1 exhibited excellent biocompatibilities for hNPMSCs *in vitro*. Moreover, RAD/SA1 had greater bioactivities for hNPMSCs than that in RADA16-I in an acidic environment *in vitro*. The relative protein secretion and gene expression (aggrecan, collagen II and SOX-9) of hNPMSCs in RAD/SA1 were significantly higher than those in RADA16-I. Furthermore, RAD/SA1 may play its biological role by inhibiting the expression of *p*-ERK through Ca^2+^-dependent *p*-ERK signaling pathways. Therefore, the functional self-assembling peptide nanofiber hydrogel designed with the short motif of Sa12b could be used as an excellent scaffold for nucleus pulposus tissue engineering. RAD/SA1 exhibits great potential applications in the regeneration of mildly degenerated nucleus pulposus.

## Data Availability

The raw data supporting the conclusion of this article will be made available by the authors, without undue reservation.
